# Letter of Dr. Charles G. Stockton, of Buffalo

**Published:** 1906-07

**Authors:** Charles G. Stockton

**Affiliations:** 436 Franklin Street


### CORRESPONDENCE.

### Letter of Dr. Charles G. Stockton, of Buffalo.

**Relating to the Rebuilding of San Francisco Medical Library—Contributions of Books are Requested—May be Sent to Librarian of the University of Buffalo.**

*Editor Buffalo Medical Journal:*

SIR:—At the late meeting of the Association of American Physicians a committee, with Dr. Charles L. Dana of New York as chairman, was appointed for the purpose of collecting books and other medical literature, to assist in rebuilding the San Francisco Medical Library, as well as to aid many needy physicians there who have lost all their books. The same committee was appointed and authorized at the late meeting of the American Medical Association.

It is urged upon the members of the profession in this region that they send all contributions at an early date in care of the librarian of the University of Buffalo, which officer has kindly consented to receive the books until they are sent on. As the local member of the committee, I shall be happy to supply any information. Will you have the kindness to insert this letter in the Buffalo Medical Journal? I shall be glad, also, if you will assist us in such other ways as you deem desirable.

CHARLES G. STOCKTON.

436 FRANKLIN STREET, JUNE 20, 1906.

---

BLOOD PRESSURE AND PULSE AS AFFECTED BY ALTITUDE.—Charles Fox Gardiner and Henry W. Hoagland describe the results obtained in conducting a large number of blood pressure determinations at Colorado Springs and at Pike's Peak, the former having an altitude of 6,000 feet, and the latter that of 14,000 feet. It was discovered that the average blood pressure of inhabitants of Colorado Springs who had been there for a year or more was slightly lower than that of dwellers at the sea level, while the pulse did not show the increased rapidity said to be present in altitude dwellers. Tests on twenty-two healthy students taken from Colorado Springs to Pike's Peak in a railway car without any muscular effort, showed that at the end of three and one half hours the blood pressure had fallen from 126 mm. to 118 mm., and the pulse rate had gone 80 to 99 per minute. The authors found a rough ratio between pulse rate and blood pressure: the more rapid the pulse, the lower the blood pressure. It was also noted that when a pulse rate was but little affected by an altitude of 14,000 feet, the blood pressure was also more constant; that cases of mountain sickness were accompanied by a fall in blood pressure and a rapid pulse rate—*Medical Record,* March 10, 1906.

